# Guideline Evaluation

**DOI:** 10.6004/jadpro.2013.4.1.8

**Published:** 2013-01-01

**Authors:** Diane Wardell

**Affiliations:** From University of Texas Health Science Center–School of Nursing, Houston, Texas

It is interesting to note that some guidelines remain relatively unchanged over many years, whereas others are relatively "fluid" in that they may or may not be consistent across groups and may also undergo considerable modification over time. This pattern may create confusion for both patients and providers. Even in those guidelines that remain relatively constant, guideline review may help to clarify potential areas of weakness and inconsistency with best practice patterns.

The process of guideline evaluation provides individuals or groups with additional information on the validity of suggestions for patient management and may ultimately lead to confidence that the guidelines will improve patient outcomes. However, individual application still requires specific knowledge about each patient’s preferences and needs. For example, although the new cervical cancer screening guidelines for women at "average" risk include a screening interval of 3 years, this may create anxiety in an individual patient who "knew" someone who had developed cervical cancer in a shorter time interval from their last Pap smear evaluation. Modification of the guideline to yearly Pap smears may then be necessary to provide the patient with an anxiety-free interval between Pap smears. In addition, this is an ideal opportunity for the advanced practice clinician to set aside time to educate and reassure the patient about her individual risk.

There are various sources for guideline evaluation, such as the Appraisal of Guidelines for Research and Evaluation instrument (AGREE Collaboration, 2001) and those by Field and Lohr (1992). The Institute of Medicine (2011) also provides recommendations that include eight standards for guideline development: transparency, management of conflict of interest, multidisciplinary group composition including patients and consumers, systematic review, evidence foundations, articulation of recommendations, external review, and updating. This discussion will include a guideline evaluation developed by Melnyk and Fineout-Overholt (2010), which was modified from Slutsky (2005).

## Questioning Guideline Development

The first question to ask is who developed the guidelines: What are their credentials and their interest in the outcome? A variety of groups and/or individuals may be considered experts on the topic and these should be included.

The next question is whether the guidelines developers represented an interdisciplinary team. This diverse composition will potentially provide a variety of viewpoints in looking at the issue and prevent a single-system approach, thus rendering the guideline broader and more holistic. Specialties should be represented, but so should the patients and consumers who are affected by the decisions, especially when dealing with long-term or chronic illnesses. This also provides an opportunity to consider different options and potential outcomes from multiple perspectives.

Because conflict of interest can interfere when funding for the development of the guideline gives the appearance of influencing decision-making, it is essential for sponsors and authors to disclose funding of any research or guideline recommendation. For example, corporate-sponsored research often has more pro-corporate findings than independent research (Bhandari et al., 2004). Publication bias (publishing only positive effects) has the potential to increase the perception of an agent’s efficacy and worth (Turner, Matthews, Linardatos, Tell, & Rosenthal, 2008). This does not necessarily prohibit the input of authors who have received corporate funding, as they may be the experts in the field. However, caution and full disclosure are necessary, especially if, for example, the supporting company’s brand is mentioned in the guideline instead of the generic name.

Developers should identify the strategy used for the guideline’s development. This would include how the literature was searched, what system was used for determining the level of evidence and how the information was rated, as well as the breadth of the review (i.e., expert testimonials, editorials, etc.). There should also be a description of how decisions were made, for example, on the inclusion and exclusion of information/studies and the individuals involved. The dates for the literature review should be logical, if not all-inclusive. For example, human papillomavirus (HPV) testing and interpretation have increased the understanding of cervical cancer screening; including studies only executed since these procedures became possible would be appropriate. Additionally, when guidelines are revised, they should identify recent and relevant studies within the current year. All guidelines should be periodically reviewed and updated to reflect current practice.

## Evaluating the Guidelines in Use

Also to be considered are the recommendations themselves. Are they clear and explicit? For example, the Cervical Cancer Screening recommendations from the U.S. Preventive Services Task Force and the American Cancer Society (including other groups) have separate guidelines that are generally consistent in their recommendations (ACOG, 2012). This promotes clarity for providers and patients.

An additional question to ask is have the guidelines been tested and evaluated in practice by peers who would be implementing them? It is important to consider whether they will make a difference in patient care and what that potential impact could be. For example, even though it is not the purpose of the cervical cancer screening guidelines to address other aspects of women’s health, they may impact women’s decisions to forego yearly clinical breast exams if they only need a Pap smear every 5 years, thus potentially missing early detection of breast cancer.

In evaluating the guidelines’ applicability and generalizability, it is important to consider the patients to whom they are directed. Is it a national or regional recommendation? Is it being made to a specific group of specialists, or is it for all practitioners? Outcomes of the recommendations should be able to be measured in relation to standard care. This step may be enhanced by electronic medical records that can follow practice procedures for individuals and groups over time.

The logistics of the recommendations also need to be considered as to resources (people, equipment, time) and practicality. They should be directive as to the setting, gender, ethnicity, and comorbidities of the patient. For example, recent cervical cancer guidelines address only the "average risk" woman and are inclusive of age. The management of women with comorbidities such as autoimmune disorders and HPV does not apply in the current guideline recommendations.

## Summary

Guidelines can provide direction and guidance for general patient management. This includes systematic evaluation of how the recommendations were derived. From this standpoint, individual patient care management decisions can be made depending on preference and risk.
